# Supplementary material for: Random walk with restart on multilayer networks: from node prioritisation to supervised link prediction and beyond
Source: BMC Bioinformatics. 2024 Feb 14;25:70. doi: 10.1186/s12859-024-05683-z (PMC10865648; doi:10.1186/s12859-024-05683-z)
Supplement: Supplementary file 1 — Additional file 1: Additional information. Supplementary Section 1. Multilayer networks. Supplementary Section 2. Node prioritisation to study human genetic diseases. Supplementary Section 3. Supervised prediction of gene-disease associations. Supplementary Section 4. Diffusion profiles comparison to unveil immune diseases similarities. Supplementary Section 5. MultiXrank workflow. Supplementary Table S1. Monoplex, multiplex and bipartite networks used in our three study cases. Of note, this tableincludes only the networks used in our analysis and does not encompass the broader non-network format information used in our studies. Supplementary Table S2. PCHi-C fragment networks characteristics. Supplementary Table S3. MultiXrank parameters used for node prioritisation in leukaemia. Supplementary Table S4. Top 10 prioritised genes for leukaemia, associated MultiXrank scores, degree and distance to seed nodes. Supplementary Table S5. Top 10 prioritised drugs for leukaemia, associated MultiXrank scores, degree and distance to seed nodes. Supplementary Table S6. Four sets of MultiXrank parameters used for node prioritisation in epilepsy. It is to note that only the λ parameter differs. Supplementary Table S7. DrugBank Categories associated to at least 7 drugs from the 41 drugs prioritised by MultiXrank (parameter set number 4, top-100) that are not prioritised by Hetionet. The first column of the table corresponds to the DrugBank Category and the second column (N ) corresponds to the number of drugs mapped to each category. It is to note that some drugs belong to more than one class. Supplementary Table S8.. MultiXrank parameters used for the supervised prediction of gene-disease associations. Supplementary Table S9. XGBoost and Random Forest models and performance metrics for predicting Gene-Diseaseassociations from DisGeNET v2.0 (2014). The models are trained and tested according to the protocol described in Supplementary Figure S5. We trained the XGBoost and Random Fo [file 12859_2024_5683_MOESM1_ESM.pdf]

Supplementary Information for

Random Walk with Restart on multilayer networks: from node prioritisation to supervised link prediction and beyond

Anthony Baptista[1, 2, *], Galadriel Brière[3], and Anaïs Baudot[4, 5, *]

[1]School of Mathematical Sciences, Queen Mary University of London, London, United Kingdom, [2] The Alan Turing Institute, London, United Kingdom, [3] Aix Marseille Univ, CNRS, I2M, Marseille, France, [4] Aix Marseille Univ, INSERM, MMG, Marseille, France, [5] Barcelona Supercomputing Center, Barcelona, Spain

* anthony.baptista@univ-amu.fr, anais.baudot@univ-amu.fr

This PDF file includes:

Figs. S1 to S9
Tables S1 to S13
SI References

Contents

1 Multilayer networks 5
  A Biological multilayer network 5
  B Hetionet multilayer network 7
  C Construction of the PCHi-C fragment and TAD networks 8
  D Addition of metabolic and regulatory networks for node prioritisation in leukaemia 9

2 Node prioritisation to study human genetic diseases 11
  A Node prioritisation in leukaemia 11
  B Node prioritisation in Epilepsy 13

3 Supervised prediction of gene-disease associations 16

4 Diffusion profiles comparison to unveil immune diseases similarities 19
  A PCHi-C and TAD experiments reflects the tree lineage of hematopoietic cells 19
  B RWR exploration of the multilayer networks including PCHi-C and TAD network layers reflects the tree lineage of the hematopoietic cells 20

5 MultiXrank workflow 29

Additional Files

Additional file 1 — Supplementary information for: Random Walk with Restart on multilayer networks: from node prioritisation to supervised link prediction and beyond. Supplementary Section 1: Multilayer networks
Supplementary Section 2: Node prioritisation to study human genetic diseases
Supplementary Section 3: Supervised prediction of gene-disease associations
Supplementary Section 4: Diffusion profiles comparison to unveil immune diseases similarities
Supplementary Section 5: MultiXrank workflow
Supplementary Table S1: Monoplex, multiplex and bipartite networks used in our three study cases. Of note, this table includes only the networks used in our analysis and does not encompass the broader non-network format information used in our studies.
Supplementary Table S2: PCHi-C fragment networks characteristics.
Supplementary Table S3: MultiXrank parameters used for node prioritisation in leukaemia.
Supplementary Table S4: Top 10 prioritised genes for leukaemia, associated MultiXrank scores, degree and distance to seed nodes.
Supplementary Table S5: Top 10 prioritised drugs for leukaemia, associated MultiXrank scores, degree and distance to seed nodes.
Supplementary Table S6: Four sets of MultiXrank parameters used for node prioritisation in epilepsy. It is to note that only the λ parameter differs.
Supplementary Table S7: DrugBank Categories associated to at least 7 drugs from the 41 drugs prioritised by MultiXrank (parameter set number 4, top-100) that are not prioritised by Hetionet. The first column of the table corresponds to the DrugBank Category and the second column (N) corresponds to the number of drugs mapped to each category. It is to note that some drugs belong to more than one class.
Supplementary Table S8: MultiXrank parameters used for the supervised prediction of gene-disease associations.
Supplementary Table S9: XGBoost and Random Forest models and performance metrics for predicting Gene-Disease associations from DisGeNET v2.0 (2014). The models are trained and tested according to the protocol described in Supplementary Figure S5. We trained the XGBoost and Random Forest models using various class weights, large weights penalising classification errors for the corresponding class (pos., for positive associations and neg., for negative associations). We report several performance metrics for each model: Balanced Accuracy (defined as the average of recall obtained on each class), F1-score and number of True Positives (TP), False Positives (FP), True Negatives (TN) and False Negative (FN). The best performing model is highlighted in grey.
Supplementary Table S10: XGBoost and Random Forest models and performance metrics for predicting Gene-Disease associations from DisGeNET v7.0 (2020). The models are the same as those presented in Supplementary Table S9, and have not been retrained for predicting DisGeNET v7.0 (2020) associations. We report several performance metrics for each model: Balanced Accuracy (defined as the average of recall obtained on each class), F1-score and number of True Positives (TP), False Positives (FP), True Negatives (TN) and False Negative (FN). The best performing model is highlighted in grey.
Supplementary Table S11: MultiXrank parameters used for diffusion profiles comparison to unveil immune diseases similarities.
Supplementary Table S12: Composition and characteristics of the immune disease clusters. Supplementary Table S13: List of the 131 immune diseases considered in this study. The first column represents the number used to identify each disease in the t-SNE projection. The second column represents the name of the disease. The third column is the UMLS identifier of the disease.

Supplementary Figure S1: Tree lineages of hematopoietic cells. MPP: Multi-Potent Progenitor ; CLP: Common Lymphoid Progenitor ; CMP: Common Myeloid Progenitor ; GMP: Granulocyte/Macrophage Progenitor ; MEP: Megakaryocyte/Erythrocyte Progenitor. Lymphoid cells and myeloid cells included in this study are indicated in red and blue, respectively.
Supplementary Figure S2: Left: Largest component of the PCHi-C fragment network built from the nB cells dataset. Right: Degree distribution of the PCHi-C fragment network defined on the left, with a zoom on low degree nodes.

Supplementary Figure S3: Representation of the multilayer network used for node prioritisation in leukaemia. The network is composed of a gene multiplex network, a drug multiplex network and their associated bipartite network. The gene multiplex network contains nodes corresponding to genes/proteins and edges corresponding to protein-protein, molecular complex, and Reactome pathway associations. The drug multiplex network contains nodes corresponding to drugs and edges corresponding to pharmacological, experimental, predicted, and clinical drug-drug interactions (Supplementary section 1.A). For the sake of clarity, the bipartite network containing the interactions connecting the two different types of nodes of the two multiplex networks is represented by the black arrow.

Supplementary Figure S4: Ictogenic properties of the 100 drugs prioritised by Hetionet and overlap with the top 100, 200, and 500 drugs prioritised by MultiXrank (parameter set 1 to 3, see Supplementary Table S6). The data on ictogenic properties has been sourced from the Hetionet study. AIGD are anti-ictogenic drugs that have a seizure suppressor effect (beige), IGD are ictogenic drugs (pink), and UKND are drugs with unknown effects (grey). The outer circle of the pie chart displays the number of AIGD, IGD, and UNKD drugs amongst the 100 drugs prioritised by Hetionet. The center of the pie chart displays the number of drugs from each category that were also prioritised by MultiXrank in the top-100 (displayed in lighter shades), top-200 (displayed in middle shade), and top-500 drugs (displayed in darker shades). The drugs prioritised in the top-500 include the drugs prioritised in the top-200, and the top-200 includes the drugs prioritised in the top-100. The white part of the pie chart corresponds to the drugs prioritised by Hetionet that are not in our MultiXrank top-500 prioritised drugs.

Supplementary Figure S5: Workflow of the random forest binary classifier. The left panel represents the training step. Here, the bipartite network connecting the gene multiplex and the disease monoplex network is built from DisGeNET v2.0 (2014). The gene-disease associations in DisGeNET v2.0 (2014) are also considered positive gene-disease associations. Negative gene-disease associations are sampled randomly. Then, MultiXrank is run using the gene and the disease nodes of each positive and negative gene-disease association, and the output scores are saved as described in the matrix *. This matrix is used with the positive and negative labels to train a random forest binary classifier. The right panel represents the test step. In this case, the true positive gene-disease associations are created from DisGeNET v7.0 (2020) and true negative gene-disease associations are sampled randomly. We next ran MultiXrank using as seeds the gene and disease nodes from each positive and negative gene-disease association, and saved the output scores in the matrix **. Finally, this ** matrix is used as an input of the previously trained random forest classifier to predict the labels of each gene-disease association. The predicted labels are then compared with the true known labels. The protocol is the same for two or three multiplex networks.

Supplementary Figure S6: 2D PCA projection of the Jaccard index similarities between the different hematopoietic cell types. Left: similarities computed on the PCHi-C fragment dataset. Right: similarities computed on the TAD dataset. Red: Lymphoid cells. Blue: Myeloid cells.

Supplementary Figure S7: 2D PCA projection of the similarity between the hematopoietic cell types. The tree lineage of hematopoietic cells is correctly found with the PCHi-C fragment, TAD and disease output scores of MultiXrank. However, the tree lineage is not recovered for the protein output scores.

Supplementary Figure S8: 2D PCA projection of hematopoietic cell similarities with respect to the integrated MultiXrank output scores effectively visualises the similarity between various hematopoietic cell types. In this projection, lymphoid cells (depicted in red) and myeloid cells (depicted in blue) exhibit a relative separation within the PCA space. Moreover, nCD4 and nCD8 cells are in close proximity to each other and relative proximity with nB cells. Furthermore, Macrophage (Mac0) and its precursor, Monocyte (Mon), appear closely situated. Additionally, Erythrocyte (Ery) and Megakaryocyte (MK), both stemming from the same progenitor, also exhibit high proximity to each other.

Supplementary Figure S9: Workflow of the MultiXrank Python package. The networks and the parameters need to be ordered as in the tree file in the left panel. This tree file is used by MultiXrank to run the RWR on the multilayer network defined in the bipartite and multiplex folders with the parameters given in the "config.yml" and "seeds.txt" files. Finally, MultiXrank returns the output scores in an output folder as shown in the right panel, each multiplex/monoplex network is associated with a ranking distribution of the nodes involved in this specific network. A Conda environment is available on GitHub (https://github.com/anthbapt/multixrank) to install all dependencies needed by MultiXrank.

1. Multilayer networks

A. Biological multilayer network.

1. Gene multiplex network. This multiplex network is composed of three layers of interactions between nodes corresponding to genes or proteins, here considered indifferently, and named according to their official gene names.
   - Complexes: A molecular complexes layer constructed from the fusion of Hu.map (2) and Corum (3), using OmniPathR (4).
   - PPI: A Protein-Protein interaction (PPI) layer corresponding to the fusion of 3 datasets: APID (homo sapiens level 2, without inter-species interactions), Hi-Union and Lit-BM (www.interactome-atlas.org/download). For APID proteins that did not match any official gene names, we used their corresponding Uniprot IDs, which was the case for 166 over 13 346 APID proteins.
   - Reactome: a pathways layer extracted from NDEx (5) and corresponding to human Reactome data (6). The network is available in NDEx: https://www.ndexbio.org/viewer/networks/4bc71515-86da-11e7-a10d-0ac135e8bacf.
2. Disease monoplex network. In this network, the nodes correspond to diseases, labelled by their UMLS identifiers, and the edges were built based on phenotypic proximities between diseases, computed considering the set of their associated phenotypes in the Human Phenotype Ontology (7). The protocol for computing those phenotypic proximities is detailed in (8).
3. Drug multiplex network. This multiplex network is composed of four layers of interactions between nodes representing drugs, identified with their DrugBank (9) names. The interaction data of this multiplex network were taken from Cheng et al. (10)[†] (first three layers) and from https://snap.stanford.edu/biodata/datasets/10001/10 ChCh-Miner.html[‡] (fourth layer).
   - Drug1[†]: Clinical drug interactions (clinically reported adverse drug-drug interactions, 14822 clinically reported adverse drug–drug interactions connecting 667 drugs.)
   - Drug2[†]: Experimental drug combinations (experimentally validated drug combinations, 737 unique pairwise drug combinations connecting 376 drugs.)
   - Drug3[†]: Predicted drug combinations (network-predicted hypertensive drug combinations, 2080 potential combinations involving 65 hypertensive drugs.)
   - Drug4[‡]: Drug-Drug interactions determined from the pharmacological effect of the action of one drug on another drug (48514 interactions involving 1514 drugs).
4. Promoter Capture Hi-C (PCHi-C) fragment monoplex networks. These networks represent the interactions between sequences of DNA. The nodes represents DNA fragments, i.e. a start and an end position on a chromosome sequence. Two fragments nodes are connected with an edge if the two DNA sequences interacts. These networks were build from the dataset provided by Javierre and co-authos (11). Importantly, we generated 8 PCHi-C fragment networks, one for each of the hematopoietic cell lines considered in our studiy. However, those 8 networks are not used in the same multilayer network, hence they are considered as monoplex networks. We detail the construction of these hematopoietic cell line specific PCHi-C fragment networks in Supplementary Section 1.C.
5. Topologically Associated Domains (TAD) monoplex networks. Thse networks represent the adjacency of TADs. The nodes represent TADs, defined by a start and end position of a chromosome sequence, are edges connects adjacent TADs. These networks were build from the dataset provided by Javierre and co-authos (11). Importantly, we generated 8 TAD networks, one for each of the hematopoietic cell lines considered in our study. However, those 8 networks are not used in the same multilayer network, hence they are considered as monoplex networks. We detail the construction of these hematopoietic cell line specific TAD networks in Supplementary Section 1.C.

Table S1. Monoplex, multiplex and bipartite networks used in our three study cases. Of note, this table includes only the networks used in our analysis and does not encompass the broader non-network format information used in our studies.

Type	Name	Association	Study 1: Node prioritisation		Study 2: Gene-Disease association prediction	Study 3: Comparison of diseases diffusion profiles
			leukaemia	Epilepsy		
Monoplex and multiplex networks	Gene multiplex	Molecular complexes membership	X		X	X
		Protein-Protein interactions				
		Pathway membership				
	Disease monoplex	Phenotypic proximity			X	X
	Drug multiplex	Clinically reported drug-drug interactions (adverse events)	X			
		Experimental drug-drug combinations				
		Predicted drug-drug combinations				
		Drug-drug pharmacological interactions				
	PCHi-C fragment monoplex	Interactions between chromatin segments for 8 hematopoietic cell types (one network per cell type)				X
	TAD monoplex	TAD adjacency in the genome for 8 hematopoeietic cell types (one network per cell type)				X
Bipartite networks	Gene-Disease DisGeNET v2.0 (2014)	Curated, inferred and literature extracted associations, scored according to their level of evidence			X	
	Gene-Disease DisGeNET v7.0 (2020)					X
	Gene-Drug	Drug-Target associations	X			
	Gene-Fragment	Genes associations to promoters in the fragment				X
	Gene-TAD	Gene included in TAD region				X
	Fragment-TAD	Fragment included in TAD region				X
Hetionet network (v1.0) (1)				X		

These various multiplex and monoplex networks can be connected to each other with bipartite networks, defined as follows:

- Gene-Disease (1-2): Gene-disease associations were downloaded from the DisGeNET database, which contains associations derived from various sources and scored according to their level of evidence (formula reported in https://www.DisGeNET.org/dbinfo). Two version of this bipartite network were used in our study:
  - Outdated: Associations derived from an outdated version of DisGeNET (v2.0, 2014) (12).
  - Updated: Associations derived from an updated version of DisGeNET (v7.0, 2020) (13).
- Gene-Drug (1-3): Drug-target associations were downloaded from several sources, and merged to obtain this bipartite network: DrugBank v5.1.8 go.drugbank.com/releases/latest, DrugCentral v10.12 drugcentral.org/download, and Cheng et al (10).
- Disease-Drug (2-3): Approved drug-disease indications were extracted from repoDB (14).
- Gene-PCHi-C Fragment (1-4): Gene-PCHi-C fragment associations were defined from the article of Javierre et al. (11), which reported genes associated to each PCHi-C fragment using the version GRCh37.p13 of the genome.
- Gene-TAD (1-5): This bipartite network establishes an association between a gene and a TAD when the gene is located within the boundaries of the TAD. Those associations were computed using gencode V19 (15), and the GRCh37.p13 version of the genome.
- PCHi-C fragment-TAD (4-5): This bipartite network connects TAD and PCHi-C fragments if the PCHi-C fragment is located within the boundaries of the TAD.

Because MultiXrank only scores nodes appearing in multiplex networks, nodes that did not share any interaction other than bipartite (i.e. with no interaction in any of the monoplex networks composing a multiplex network) were artificially added to their corresponding multiplex network by creating self-interactions (i.e. self-loop). This intervention is purely technical and does not have effects on MultiXrank output scores.

B. Hetionet multilayer network.

The Hetionet network is a gene-, compound- and disease-centric network constructed by integrating knowledge and experimental findings from millions of biomedical research publications (1).

Hetionet is composed of three layers:

- Gene multiplex network composed of three layers: Gene–covaries–Gene (GcG), Gene–interacts–Gene (GiG), Gene-regulates-Gene (GrG)
- Disease monoplex network: Disease–resembles–Disease (DrD)
- Compound monoplex network: Compound–resembles–Compound (CrC)

Gene, disease and compounds nodes are connected by several bipartite networks:

- Gene-Disease: Union of three types of interaction, Disease–associates–Gene (DaG), Disease–downregulates–Gene (DdG), Disease–upregulates–Gene (DuG)
- Compound-Disease: Union of two types of interaction, Compound–palliates–Disease (CpD), Compound–treats–Disease (CtD)
- Compound-Gene: Union of two types of interaction, Compound–downregulates–Gene (CdG), Compound–upregulates–Gene (CuG)

Hetionet also consider additional bipartite interactions for genes, compounds and diseases:

- Gene-Pathway: Gene–participates–Pathway (GpPW)
- Gene-Biological Process: Gene–participates–Biological Process (GpBP)
- Gene-Molecular Function: Gene–participates–Molecular Function (GpMF)
- Gene-Cellular Component: Gene–participates–Cellular Component (GpCC)
- Anatomy Part-Gene: Merged of three layers, Anatomy–downregulates–Gene (AdG), Anatomy–expresses–Gene (AeG), Anatomy–upregulates–Gene (AuG)
- Disease-Anatomy part: Disease–localises–Anatomy (DlA)
- Disease-Symptom: Disease–presents–Symptom (DpS)
- Compound-Side effect: Compound–causes–Side Effect (CcSE)
- Pharmalogic classes-Compound: Pharmacologic Class–includes–Compound (PCiC)

Since only bipartite interactions are considered for many node types (Pathway, Biological Process, Anatomy parts, Symptoms, Side Effects and Pharmacologic class), for these, we created artificial self-loops to match MultiXrank formalism. This intervention is purely technical and does not have effects on MultiXrank output scores.

C. Construction of the PCHi-C fragment and TAD networks.

The PCHi-C fragment and TAD networks were generated for various hematopoietic cell lines, based on the datasets generated by Javierre et al. (11). Eight hematopoietic cell lines were considered: Erythrocytes (Ery), Macrophages (Mac0), Megakaryocytes (MK), Monocytes (Mon), Naive B Cells (nB), Naive CD4+ T Cells (nCD4), Naive CD8+ T Cells (nCD8) and Neutrophils (Neu). The corresponding tree lineage is reported in Supplementary Figure S1.

The datasets, originally produced and processed by Javierre and co-authors using the CHICAGO pipeline (16) to identify significant interactions (score > 5) in various hematopoietic cell lines. The processed data are represented below:

fragments 1			fragments 2			Score
chr	start	end	chr	start	end	
	⋮			⋮		⋮
	⋮			⋮		⋮
	⋮			⋮		⋮

Using these results, we were able to create a network for each hematopoietic cell line, containing the PCHi-C fragment interactions identified by Javierre et al.

In their study, Javierre et al. also used the directionality index score (17) to identify TADs in the 8 hematopoietic cell lines. The dataset is represented below:

TADs		
chr	start	end
	⋮	
	⋮	
	⋮	

Building upon these results, we were able to construct the TAD networks by connecting adjacent TADs.

We report several features of the obtained PCHi-C fragment networks in Supplementary Table S2 and in Supplementary Figure S2.

Table S2. PCHi-C fragment networks characteristics

Cellular Type	Naive B cells (nB)	Erythroblasts (Ery)	Macrophages M0 (Mac0)	Monocytes (Mon)
# Nodes	101973	90184	109566	96443
# Edges	192104	152617	182250	167275
# Isolated Nodes	0	0	0	0
# Self Loops	0	0	0	0
Density	3.7E-05	3.8E-05	3E-05	3.6E-05
Avg. Clustering Coeff.	0.152	0.092	0.094	0.14
Avg. Degree	3.768	3.385	3.327	3.469

Cellular Type	Naive CD4+ T cells (nCD4)	Naive CD8+ T cells (nCD8)	Neutrophils (Neu)	Megakaryocytes (MK)
# Nodes	106849	108016	81244	98159
# Edges	212106	218293	143679	152347
# Isolated Nodes	0	0	0	0
# Self Loops	0	0	0	0
Density	3.7E-05	3.7E-05	4.4E-05	3.2E-05
Avg. Clustering Coeff.	0.188	0.18	0.151	0.091
Avg. Degree	3.97	4.042	3.537	3.104

D. Addition of metabolic and regulatory networks for node prioritisation in leukaemia.

Please, note that we provide a supplementary analysis for node prioritisation in leukaemia as a Jupyter-Notebook tutorial available on our Github (https://github.com/galadrielbriere/ApplicationsMultiXrank). This Jupyter-Notebook includes the construction of a fourth layer in the gene multiplex network, featuring directed interactions representing Transcription Factors and their regulated targets in leukaemia downloaded from the GRNdb database (18, 19). Additionally, we introduced a new monoplex network of metabolic reactions, utilising data sourced from the Human-GEM database (20).

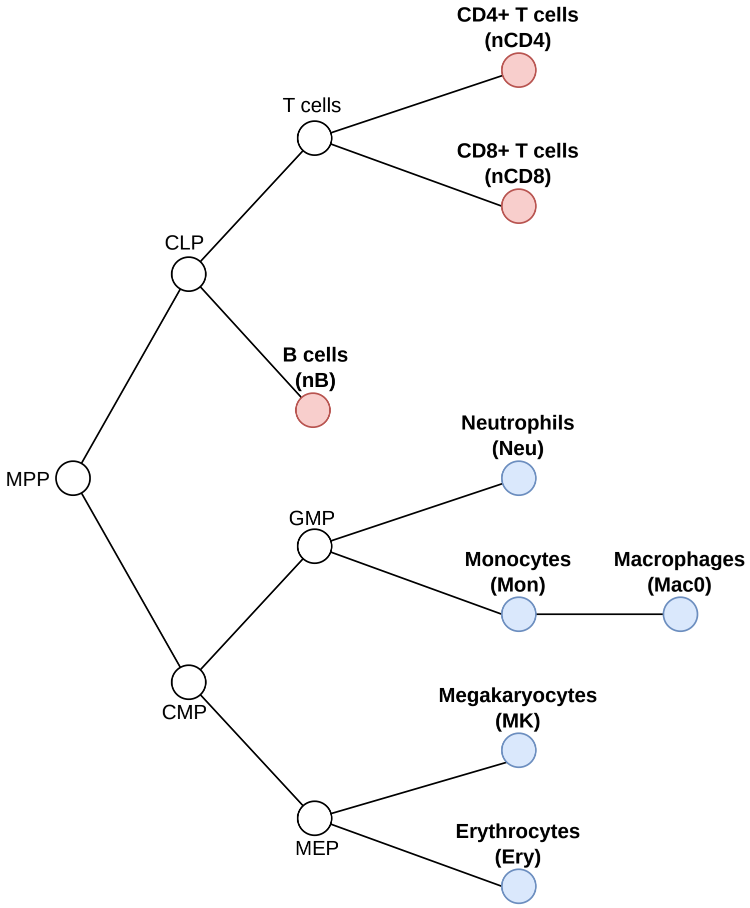

Fig. S1. Tree lineages of hematopoietic cells. MPP: Multi-Potent Progenitor ; CLP: Common Lymphoid Progenitor ; CMP: Common Myeloid Progenitor ; GMP: Granulocyte/Macrophage Progenitor ; MEP: Megakaryocyte/Erythrocyte Progenitor. Lymphoid cells and myeloid cells included in this study are indicated in red and blue, respectively.

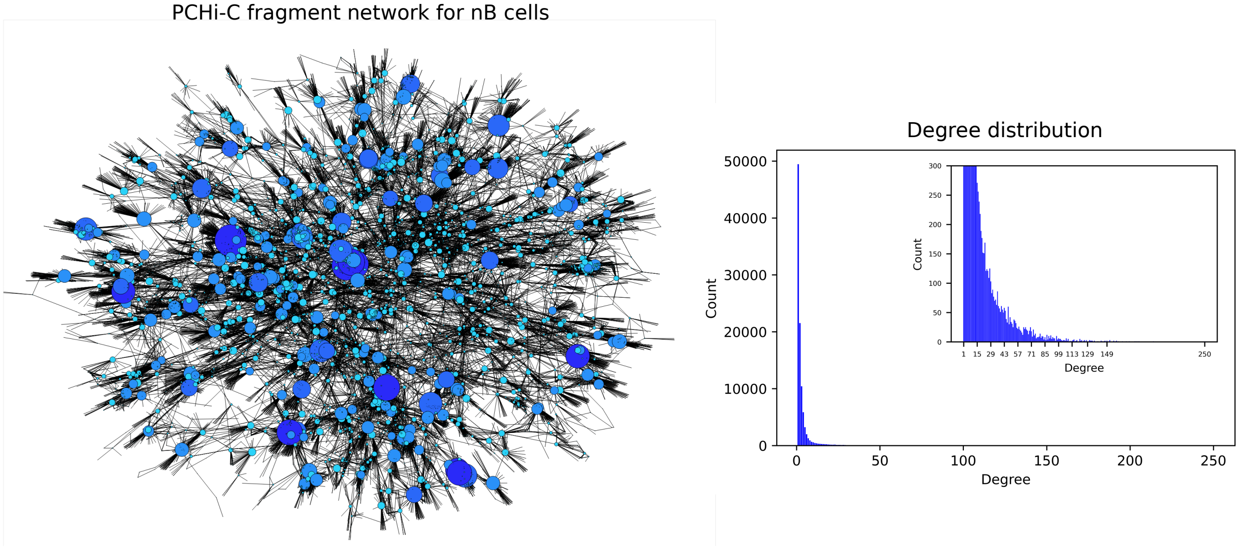

Fig. S2. Left: Largest component of the PCHi-C fragment network built from the nB cells dataset. Right: Degree distribution of the PCHi-C fragment network defined on the left, with a zoom on low degree nodes.

2. Node prioritisation to study human genetic diseases

A. Node prioritisation in leukaemia.

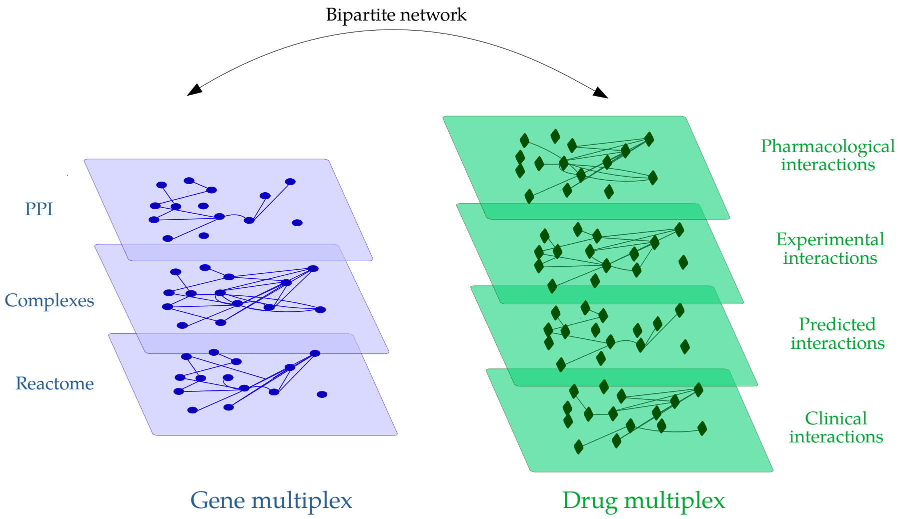

Fig. S3. Representation of the multilayer network used for node prioritisation in leukaemia. The network is composed of a gene multiplex network, a drug multiplex network and their associated bipartite network. The gene multiplex network contains nodes corresponding to genes/proteins and edges corresponding to protein-protein, molecular complex, and Reactome pathway associations. The drug multiplex network contains nodes corresponding to drugs and edges corresponding to pharmacological, experimental, predicted, and clinical drug-drug interactions (Supplementary section 1.A). For the sake of clarity, the bipartite network containing the interactions connecting the two different types of nodes of the two multiplex networks is represented by the black arrow.

Top-10 prioritised genes and their known or suspected relations with leukaemia:

1. CYP3A4 is known to be implicated in leukaemia (21).
2. FNTB is the target of Tipifarnib (our seed drug) according to the DrugBank database.
3. RAF1 mutations were identified in patients with Noonan syndrome (22), which is a well-known risk factor for leukaemia (23).
4. RASGRP1 has been linked with leukaemia (24).
5. DGKZ knockdown can induce apoptosis in human Acute Myeloid leukaemia (AML) HL-60 cells through the MAPK/survivin/caspase pathway (25).
6. RIN1 plays a role in the maintenance of the abnormal RTK signaling in chronic myeloid leukaemia (26).
7. BRAF mutations were identified in AML patients with monocytic differentiation (27).
8. RASA1 is involved in several types of cancer, including leukaemia (28).
9. AURKA: A significant association between over-expression of AURKA and cytogenetic abnormalities was found in AML patients (29).
10. ARAF: A study found ARAF gene mutation in MOLT-4 leukaemia cell line (30).

Top-10 prioritised drugs and their known or suspected relations with leukaemia:

1. DB00637 (Astemizole) belongs to a drug class that was validated for its activities against human primary AML samples (31).

2. DB01380 (Cortisone acetate) is associated to acute leukaemia, according to the DrugBank database.
3. DB00630 (Alendronate): Some preliminary experiments show a benefit for the treatment of osteopenia/osteoporosis with Alendronate in children with acute lymphoblastic leukaemia (32).
4. DB00398 (Sorafenib) is a drug used for the treatment of advanced renal cell carcinoma, and some studies mention its impact on leukaemia (33).
5. DB00399 (Zoledronic acid) is a drug used to prevent skeletal fractures in patients with cancers such as multiple myeloma and prostate cancer, and some studies show interesting links with leukaemia (34).
6. DB00773 (Etoposide) is used for first-line treatment in patients with small-cell lung cancer. It is also used to treat other malignancies such as lymphoma or nonlymphocytic leukaemia, according to DrugBank.
7. DB01254 (Dasatinib) is used in patients with Chronic Myelogenous leukaemia, according to DrugBank.
8. DB01268 (Sunitinib): A phase I/II study of sunitinib and intensive chemotherapy for AML patients with FLT3 mutations has been achieved with encouraging results (35).
9. DB06589 (Pazopanib): This drug is in clinical trial phase II for patients with AML (36).
10. DB00530 (Erlotinib) is an inhibitor of the epidermal growth factor receptor (EGFR) tyrosine kinase that is used in the treatment of several types of cancer, including leukaemia (37).

Table S3. MultiXrank parameters used for node prioritisation in leukaemia.

r	δ	τ	λ	η
0.7	$\begin{bmatrix} 1/2 & 1/2 \end{bmatrix}$	$\begin{bmatrix} 1/3 & 1/3 & 1/3 & 0 \\ 1/4 & 1/4 & 1/4 & 1/4 \end{bmatrix}$	$\begin{bmatrix} 1/2 & 1/2 \\ 1/2 & 1/2 \end{bmatrix}$	$\begin{bmatrix} 1/2 & 1/2 \end{bmatrix}$

Table S4. Top 10 prioritised genes for leukaemia, associated MultiXrank scores, degree and distance to seed nodes.

Genes	Scores	Degree	Distance to HRAS	Distance to Tipifarnib
CYP3A4	0.010934	5	3	1
FNTB	0.010690	16	3	1
RAF1	0.001010	155	1	2
RASGRP1	0.000984	142	1	3
DGKZ	0.000780	17	1	4
RIN1	0.000746	55	1	3
BRAF	0.000237	168	1	2
RASA1	0.000215	83	1	3
AURKA	0.000207	178	1	2
ARAF	0.000205	54	1	3

Please, note that we provide a supplementary analysis for node prioritisation in leukaemia including regulatory and metabolic networks, in the form of a Jupyter-Notebook tutorial available on our Github (https://github.com/galadrielbriere/ApplicationsMultiXrank). This Jupyter-Notebook includes an analysis and interpretation of the novel results.

Table S5. Top 10 prioritised drugs for leukaemia, associated MultiXrank scores, degree and distance to seed nodes.

Drugs	Scores	Degree	Distance to HRAS	Distance to Tipifarnib
DB00637	0.000357	26	2	2
DB01380	0.000349	69	4	2
DB00630	0.000326	32	4	2
DB00398	0.000267	108	2	1
DB00399	0.000195	24	3	1
DB00773	0.000191	80	3	1
DB01254	0.000163	435	2	2
DB01268	0.000144	157	1	2
DB06589	0.000100	171	2	3
DB00530	0.000085	95	2	2

B. Node prioritisation in Epilepsy.

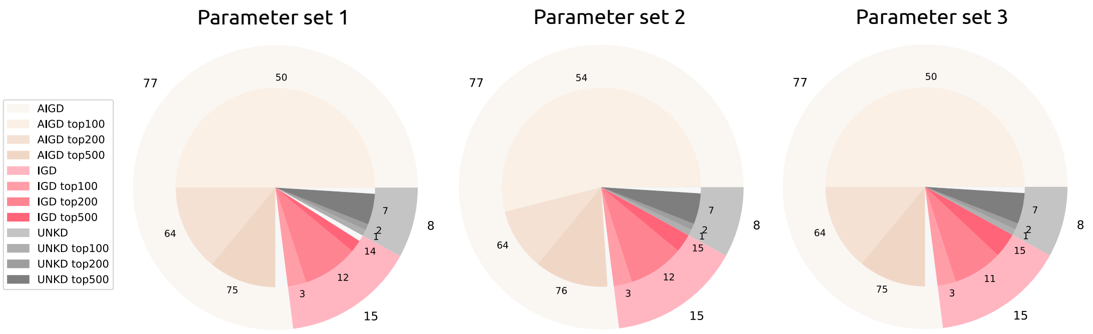

Fig. S4. Ictogenic properties of the 100 drugs prioritised by Hetionet and overlap with the top 100, 200, and 500 drugs prioritised by MultiXrank (parameter set 1 to 3, see Supplementary Table S6). The data on ictogenic properties has been sourced from the Hetionet study. AIGD are anti-ictogenic drugs that have a seizure suppressor effect (beige), IGD are ictogenic drugs (pink), and UKND are drugs with unknown effects (grey). The outer circle of the pie chart displays the number of AIGD, IGD, and UNKD drugs amongst the 100 drugs prioritised by Hetionet. The center of the pie chart displays the number of drugs from each category that were also prioritised by MultiXrank in the top-100 (displayed in lighter shades), top-200 (displayed in middle shade), and top-500 drugs (displayed in darker shades). The drugs prioritised in the top-500 include the drugs prioritised in the top-200, and the top-200 includes the drugs prioritised in the top-100. The white part of the pie chart corresponds to the drugs prioritised by Hetionet that are not in our MultiXrank top-500 prioritised drugs.

Table S6. Four sets of MultiXrank parameters used for node prioritisation in epilepsy. It is to note that only the λ parameter differs.

r	δ	τ	λ	η
0.7	$\begin{bmatrix} 1/2 & 0 & 0 & 0 & 0 & 0 & 0 & 0 & 0 \end{bmatrix}$	$\begin{bmatrix} 1/3 & 1/3 & 1/3 & 0 \\ 1 & 0 & 0 & 0 \\ 1 & 0 & 0 & 0 \\ 1 & 0 & 0 & 0 \\ 1 & 0 & 0 & 0 \\ 1 & 0 & 0 & 0 \\ 1 & 0 & 0 & 0 \\ 1 & 0 & 0 & 0 \\ 1 & 0 & 0 & 0 \end{bmatrix}$	$\begin{bmatrix} 1/9 & 1/9 & 1/9 & 1/9 & 1/9 & 1/9 & 1/9 & 1/9 & 1/9 \\ 1/9 & 1/9 & 1/9 & 1/9 & 1/9 & 1/9 & 1/9 & 1/9 & 1/9 \\ 1/9 & 1/9 & 1/9 & 1/9 & 1/9 & 1/9 & 1/9 & 1/9 & 1/9 \\ 1/9 & 1/9 & 1/9 & 1/9 & 1/9 & 1/9 & 1/9 & 1/9 & 1/9 \\ 1/9 & 1/9 & 1/9 & 1/9 & 1/9 & 1/9 & 1/9 & 1/9 & 1/9 \\ 1/9 & 1/9 & 1/9 & 1/9 & 1/9 & 1/9 & 1/9 & 1/9 & 1/9 \\ 1/9 & 1/9 & 1/9 & 1/9 & 1/9 & 1/9 & 1/9 & 1/9 & 1/9 \\ 1/9 & 1/9 & 1/9 & 1/9 & 1/9 & 1/9 & 1/9 & 1/9 & 1/9 \\ 1/9 & 1/9 & 1/9 & 1/9 & 1/9 & 1/9 & 1/9 & 1/9 & 1/9 \end{bmatrix}$	$\begin{bmatrix} 0 & 1 & 0 & 0 & 0 & 0 & 0 & 0 & 0 \end{bmatrix}$

Parameter set 1

r	δ	τ	λ	η
0.7	$\begin{bmatrix} 1/2 & 0 & 0 & 0 & 0 & 0 & 0 & 0 & 0 \end{bmatrix}$	$\begin{bmatrix} 1/3 & 1/3 & 1/3 & 0 \\ 1 & 0 & 0 & 0 \\ 1 & 0 & 0 & 0 \\ 1 & 0 & 0 & 0 \\ 1 & 0 & 0 & 0 \\ 1 & 0 & 0 & 0 \\ 1 & 0 & 0 & 0 \\ 1 & 0 & 0 & 0 \\ 1 & 0 & 0 & 0 \end{bmatrix}$	$\begin{bmatrix} 0 & 1/9 & 1/9 & 1/9 & 1/9 & 1/9 & 1/9 & 1/9 & 1/9 \\ 1/9 & 0 & 1/9 & 1/9 & 1/9 & 1/9 & 1/9 & 1/9 & 1/9 \\ 2/9 & 2/9 & 1/9 & 2/9 & 2/9 & 2/9 & 2/9 & 2/9 & 2/9 \\ 1/9 & 1/9 & 1/9 & 0 & 1/9 & 1/9 & 1/9 & 1/9 & 1/9 \\ 1/9 & 1/9 & 1/9 & 1/9 & 0 & 1/9 & 1/9 & 1/9 & 1/9 \\ 1/9 & 1/9 & 1/9 & 1/9 & 1/9 & 0 & 1/9 & 1/9 & 1/9 \\ 1/9 & 1/9 & 1/9 & 1/9 & 1/9 & 1/9 & 0 & 1/9 & 1/9 \\ 1/9 & 1/9 & 1/9 & 1/9 & 1/9 & 1/9 & 1/9 & 0 & 1/9 \\ 1/9 & 1/9 & 1/9 & 1/9 & 1/9 & 1/9 & 1/9 & 1/9 & 0 \end{bmatrix}$	$\begin{bmatrix} 0 & 1 & 0 & 0 & 0 & 0 & 0 & 0 & 0 \end{bmatrix}$

Parameter set 2

r	δ	τ	λ	η
0.7	$\begin{bmatrix} 1/2 & 0 & 0 & 0 & 0 & 0 & 0 & 0 & 0 \end{bmatrix}$	$\begin{bmatrix} 1/3 & 1/3 & 1/3 & 0 \\ 1 & 0 & 0 & 0 \\ 1 & 0 & 0 & 0 \\ 1 & 0 & 0 & 0 \\ 1 & 0 & 0 & 0 \\ 1 & 0 & 0 & 0 \\ 1 & 0 & 0 & 0 \\ 1 & 0 & 0 & 0 \\ 1 & 0 & 0 & 0 \end{bmatrix}$	$\begin{bmatrix} 0 & 1/18 & 1/18 & 1/18 & 1/18 & 1/18 & 1/18 & 1/18 & 1/18 \\ 1/9 & 1/9 & 1/9 & 1/9 & 1/9 & 1/9 & 1/9 & 1/9 & 1/9 \\ 2/9 & 3/18 & 3/18 & 5/18 & 5/18 & 5/18 & 5/18 & 5/18 & 5/18 \\ 1/9 & 1/9 & 1/9 & 0 & 1/9 & 1/9 & 1/9 & 1/9 & 1/9 \\ 1/9 & 1/9 & 1/9 & 1/9 & 0 & 1/9 & 1/9 & 1/9 & 1/9 \\ 1/9 & 1/9 & 1/9 & 1/9 & 1/9 & 0 & 1/9 & 1/9 & 1/9 \\ 1/9 & 1/9 & 1/9 & 1/9 & 1/9 & 1/9 & 0 & 1/9 & 1/9 \\ 1/9 & 1/9 & 1/9 & 1/9 & 1/9 & 1/9 & 1/9 & 0 & 1/9 \\ 1/9 & 1/9 & 1/9 & 1/9 & 1/9 & 1/9 & 1/9 & 1/9 & 0 \end{bmatrix}$	$\begin{bmatrix} 0 & 1 & 0 & 0 & 0 & 0 & 0 & 0 & 0 \end{bmatrix}$

Parameter set 3

r	δ	τ	λ	η
0.7	$\begin{bmatrix} 1/2 & 0 & 0 & 0 & 0 & 0 & 0 & 0 & 02 \end{bmatrix}$	$\begin{bmatrix} 1/3 & 1/3 & 1/3 & 0 \\ 1 & 0 & 0 & 0 \\ 1 & 0 & 0 & 0 \\ 1 & 0 & 0 & 0 \\ 1 & 0 & 0 & 0 \\ 1 & 0 & 0 & 0 \\ 1 & 0 & 0 & 0 \\ 1 & 0 & 0 & 0 \\ 1 & 0 & 0 & 0 \end{bmatrix}$	$\begin{bmatrix} 0 & 1/9 & 1/9 & 1/9 & 1/9 & 1/9 & 1/9 & 1/9 & 1/9 \\ 1/9 & 0 & 1/9 & 1/9 & 1/9 & 1/9 & 1/9 & 1/9 & 1/9 \\ 3/9 & 3/9 & 2/9 & 3/9 & 3/9 & 3/9 & 3/9 & 3/9 & 3/9 \\ 1/9 & 1/9 & 1/9 & 0 & 1/9 & 1/9 & 1/9 & 1/18 & 1/18 \\ 1/9 & 1/9 & 1/9 & 1/9 & 0 & 1/9 & 1/9 & 1/9 & 1/9 \\ 1/9 & 1/9 & 1/9 & 1/9 & 1/9 & 0 & 1/9 & 1/9 & 1/9 \\ 1/9 & 1/9 & 1/9 & 1/9 & 1/9 & 1/9 & 0 & 1/9 & 1/9 \\ 1/18 & 1/18 & 1/18 & 1/18 & 1/18 & 1/18 & 1/18 & 0 & 1/18 \\ 1/18 & 1/18 & 1/18 & 1/18 & 1/18 & 1/18 & 1/18 & 1/18 & 0 \end{bmatrix}$	$\begin{bmatrix} 0 & 1 & 0 & 0 & 0 & 0 & 0 & 0 & 0 \end{bmatrix}$

Parameter set 4

Table S7. DrugBank Categories associated to at least 7 drugs from the 41 drugs prioritised by MultiXrank (parameter set number 4, top-100) that are not prioritised by Hetionet. The first column of the table corresponds to the DrugBank Category and the second column (N) corresponds to the number of drugs mapped to each category. It is to note that some drugs belong to more than one class.

**Drugs class**	**N**	**Drugs class**	**N**	**Drugs class**	**N**
Cytochrome P-450 Substrates	24	Peripheral Nervous System Agents	10	Agents causing hyperkalemia	8
Agents that produce hypertension	18	Antimigraine Preparations	10	Cytochrome P-450 CYP2C19 Substrates	7
Central Nervous System Depressants	18	Cardiovascular Agents	10	Cytochrome P-450 CYP3A4 Inhibitors	7
Neurotransmitter Agents	17	Sensory System Agents	10	Enzyme Inhibitors	7
Analgesics	17	P-glycoprotein substrates	9	Biogenic Amines	7
Heterocyclic Compounds Fused-Ring	15	Drugs that are Mainly Renally Excreted	9	Biogenic Monoamines	7
Cytochrome P-450 Enzyme Inhibitors	14	Serotonin Modulators	9	Selective Serotonin 5-HT1 Receptor Agonists	7
Cytochrome P-450 CYP3A4 Substrates	14	Serotonin Receptor Agonists	9	Selective Serotonin Agonists	7
Amines	12	Serotonin 5-HT1 Receptor Agonists	9	Serotonin 1b Receptor Agonists	7
Cytochrome P-450 CYP1A2 Substrates	12	Cytochrome P-450 CYP2D6 Substrates	9	Serotonin 1d Receptor Agonists	7
Serotonergic Drugs	11	Cytochrome P-450 CYP2C9 Substrates	8	Triptans	7
Antidepressive Agents	10	Indoles	8	P-glycoprotein inhibitors	7
Serotonin Agents	10	Analgesics Non-Narcotic	8		

3. Supervised prediction of gene-disease associations

The classifiers were trained on MultiXrank scores generated from exploring the multilayer network, taking as seeds the gene and disease nodes considered in the association. We detail the procedure bellow.

1. **Creation of the training dataset from DisGeNET v2.0 (2014) associations**
   Gene-disease associations were obtained from an outdated version of DisGeNET (v2.0, 2014). We filtered out associations with scores below 0,5 in order to remove associations that had insufficient support in the literature and obtained 1914 gene-disease associations. These associations constitute our positive dataset. On the other hand, we generated a negative dataset by randomly picking 3828 pairs of gene and disease nodes that are not considered associated in DisGeNET v2.0 (2014). We selected twice as many negative associations than positive associations in the training set in order to account for class imbalance, which is likely to occur in real settings. We thus obtained 5742 labelled associations. We further split this dataset into a training and test set, keeping 30% of the associations for evaluating the performance of the models to predict DisGeNET v2.0 (2014) associations.

2. **Running MultiXrank for all associations**
   For each positive and negative gene-disease association defined in the training dataset, we used both the gene and the disease nodes as seeds when running MultiXrank. For positive associations, we also removed the bipartite edge between the two seeds before running MultiXrank. For each pair of seed (i.e. positive or negative association), we ran MultiXrank on the multilayer network with the parameters defined in Supplementary Table S8. These scores encode the similarity of all the nodes of the multilayer network with respect to the seeds, which we can use to train a classifier for predicting gene-disease associations.

3. **Training binary classifiers from MultiXrank scores**
   The output scores obtained with MultiXrank using the positive and negative gene-disease associations as seeds were used to train Random Forest and XGBoost classifiers. For both classifiers, we used various class weights, which are often used in imbalanced classifications problems for penalising classification errors on the minority class. The class weights used to train the classifiers are described in Supplementary Table S9, in which we also report the performances of each models in predicting the associations from DisGeNET v2.0 (2014) that were kept as a test set.

4. **Creation of the test dataset from DisGeNET v7.0 (2020) associations**
   To evaluate weather novel associations from DisGeNET v7.0 (2020) could have been predicted based on MultiXrank scores obtained using DisGeNET v2.0 (2014) associations only, we create a test dataset containing positive and negative gene-disease associations from DisGeNET v7.0 (2020). The positive associations were extracted from DisGeNET v7.0 (selecting associations above a threshold equal to 0.5) and the negative associations were generated by randomly selecting pairs of gene and disease that are not considered associated in DisGeNET v2.0. We obtained 7218 novel positive gene-disease associations, and randomly selected twice as many (i.e. 14 436) negative associations. We made sure all selected positive and negative associations were considering genes and diseases that were already present in the gene and disease layers of the multilayer network.

5. **Evaluation of the random forest binary classifier on the test dataset from DisGeNET v7.0 (2020) associations**
   We ran MultiXrank using as seeds the gene and disease nodes of each positive and negative gene-disease association of the test dataset, using the same parameters used for obtaining MultiXrank scores for the training dataset (Supplementary Table S8). We used MultiXrank output scores as input of the previously computed Random Forest and XGBoost models (trained on the data obtained with the gene-disease association of DisGeNET v2.0 (2014)) and predicted their label. Finally, we compared the predicted labels to the true labels. We report the results for each model in Supplementary Table S10.

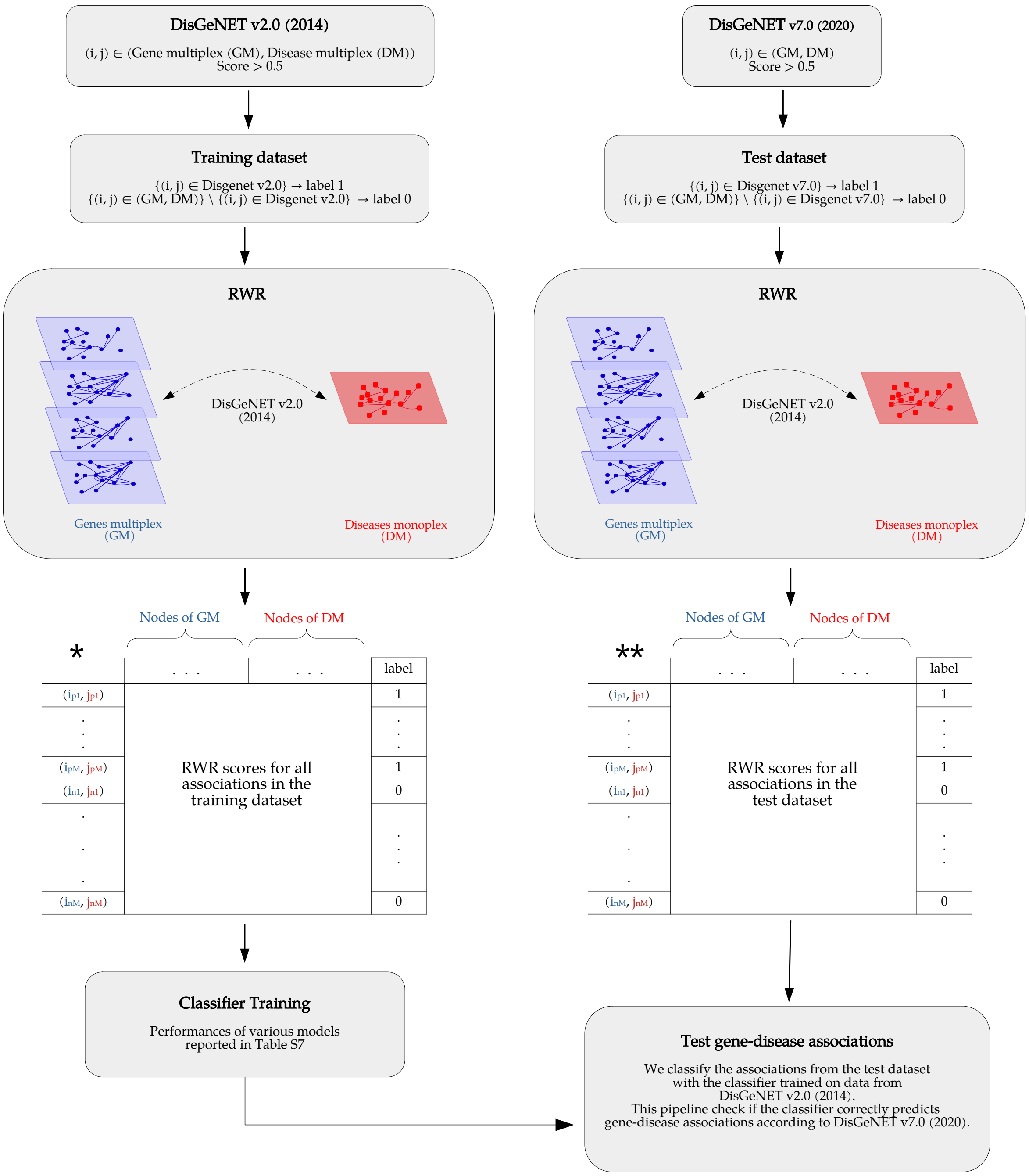

Fig. S5. Workflow of the random forest binary classifier. The left panel represents the training step. Here, the bipartite network connecting the gene multiplex and the disease monoplex network is built from DisGeNET v2.0 (2014). The gene-disease associations in DisGeNET v2.0 (2014) are also considered positive gene-disease associations. Negative gene-disease associations are sampled randomly. Then, MultiXrank is run using the gene and the disease nodes of each positive and negative gene-disease association, and the output scores are saved as described in the matrix *. This matrix is used with the positive and negative labels to train a random forest binary classifier. The right panel represents the test step. In this case, the true positive gene-disease associations are created from DisGeNET v7.0 (2020) and true negative gene-disease associations are sampled randomly. We next ran MultiXrank using as seeds the gene and disease nodes from each positive and negative gene-disease association, and saved the output scores in the matrix **. Finally, this ** matrix is used as an input of the previously trained random forest classifier to predict the labels of each gene-disease association. The predicted labels are then compared with the true known labels. The protocol is the same for two or three multiplex networks.

Table S8. MultiXrank parameters used for the supervised prediction of gene-disease associations.

r	δ	τ	λ	η
0.7	$\begin{bmatrix} 1/2 & 0 \end{bmatrix}$	$\begin{bmatrix} 1/3 & 1/3 & 1/3 \\ 1 & 0 & 0 \end{bmatrix}$	$\begin{bmatrix} 1/2 & 1/2 \\ 1/2 & 1/2 \end{bmatrix}$	$\begin{bmatrix} 1/2 & 1/2 \end{bmatrix}$

Table S9. XGBoost and Random Forest models and performance metrics for predicting Gene-Disease associations from DisGeNET v2.0 (2014). The models are trained and tested according to the protocol described in Supplementary Figure S5. We trained the XGBoost and Random Forest models using various class weights, large weights penalising classification errors for the corresponding class (pos., for positive associations and neg., for negative associations). We report several performance metrics for each model: Balanced Accuracy (defined as the average of recall obtained on each class), F1-score and number of True Positives (TP), False Positives (FP), True Negatives (TN) and False Negative (FN). The best performing model is highlighted in grey.

Classifier	Class Weight Neg.	Class Weight Pos.	Balanced Accuracy	F1 Score	TP	FP	TN	FN
XGBoost	0.1	0.9	0.853	0.792	508	197	948	70
XGBoost	0.2	0.8	0.844	0.787	481	164	981	97
XGBoost	0.3	0.7	0.848	0.795	472	138	1007	106
XGBoost	0.4	0.6	0.838	0.785	452	121	1024	126
XGBoost	0.5	0.5	0.822	0.768	421	97	1048	157
XGBoost	0.6	0.4	0.816	0.762	412	92	1053	166
XGBoost	0.7	0.3	0.807	0.751	390	70	1075	188
XGBoost	0.8	0.2	0.801	0.745	377	57	1088	201
XGBoost	0.9	0.1	0.767	0.695	331	44	1101	247
Random Forest	0.1	0.9	0.740	0.651	320	85	1060	258
Random Forest	0.2	0.8	0.749	0.666	319	61	1084	259
Random Forest	0.3	0.7	0.739	0.649	312	71	1074	266
Random Forest	0.4	0.6	0.772	0.701	347	65	1080	231
Random Forest	0.5	0.5	0.769	0.697	348	73	1072	230
Random Forest	0.6	0.4	0.784	0.718	369	81	1064	209
Random Forest	0.7	0.3	0.792	0.729	386	95	1050	192
Random Forest	0.8	0.2	0.812	0.756	411	99	1046	167
Random Forest	0.9	0.1	0.818	0.762	422	108	1037	156

Table S10. XGBoost and Random Forest models and performance metrics for predicting Gene-Disease associations from DisGeNET v7.0 (2020). The models are the same as those presented in Supplementary Table S9, and have not been retrained for predicting DisGeNET v7.0 (2020) associations. We report several performance metrics for each model: Balanced Accuracy (defined as the average of recall obtained on each class), F1-score and number of True Positives (TP), False Positives (FP), True Negatives (TN) and False Negative (FN). The best performing model is highlighted in grey.

Model	Class Weight Neg.	Class Weight Pos.	Balanced Accuracy	F1 Score	TP	FP	TN	FN
XGBoost	0.1	0.9	0.642	0.530	4026	3950	10486	3192
XGBoost	0.2	0.8	0.633	0.509	3631	3423	11013	3587
XGBoost	0.3	0.7	0.633	0.501	3402	2972	11464	3816
XGBoost	0.4	0.6	0.627	0.484	3140	2613	11823	4078
XGBoost	0.5	0.5	0.622	0.468	2901	2274	12162	4317
XGBoost	0.6	0.4	0.616	0.449	2658	1971	12465	4560
XGBoost	0.7	0.3	0.613	0.438	2525	1785	12651	4693
XGBoost	0.8	0.2	0.606	0.415	2279	1498	12938	4939
XGBoost	0.9	0.1	0.598	0.388	2030	1218	13218	5188
Random Forest	0.1	0.9	0.566	0.352	1975	2035	12401	5243
Random Forest	0.2	0.8	0.576	0.361	1972	1745	12691	5246
Random Forest	0.3	0.7	0.578	0.367	2029	1817	12619	5189
Random Forest	0.4	0.6	0.583	0.381	2155	1925	12511	5063
Random Forest	0.5	0.5	0.594	0.404	2309	1914	12522	4909
Random Forest	0.6	0.4	0.600	0.422	2493	2102	12334	4725
Random Forest	0.7	0.3	0.609	0.446	2732	2307	12129	4486
Random Forest	0.8	0.2	0.615	0.462	2932	2556	11880	4286
Random Forest	0.9	0.1	0.618	0.474	3129	2847	11589	4089

4. Diffusion profiles comparison to unveil immune diseases similarities

A. PCHi-C and TAD experiments reflects the tree lineage of hematopoietic cells.

Before subjecting each hematopoietic cell line-specific multilayer network to analysis using MultiXrank our objective was to ascertain whether the PCHi-C and TAD datasets could effectively distinguish between the different hematopoietic cell lines. The hierarchical lineage of hematopoietic cells is visually represented in Supplementary Figure S1. The tree lineage is composed of two branches: the first branch encompasses lymphoid cells, while the second branch encompasses myeloid cells.

The lymphoid cells branch is further divided into two sub-branches: the first one contains the B-cells with the naive B cells (nB), and the second one contains the T-cells with the Naive CD4+ T cells (nCD4) and the Naive CD8+ T cells (nCD8).

The myeloid cells branch is also further divided into two other branches: the first one contains the Monocytes (Mon) and the Neutrophils (Neu), and the second one contains the Megakaryocytes (MK), and the Erythroblasts (Ery). The Macrophages M0 (Mac0) are differentiated from monocytes (38).

To assess the ability of the PCHi-C and TAD datasets to differentiate among these diverse cell types, we first defined a similarity measure to evaluate the similarity of cell lines according to the PCHi-C and TAD datasets. We used the Jaccard index as a similarity metric, to evaluate the proportion of shared PCHi-C fragments and TAD between hematopoietic cells:

$$J_{i,j} = \frac{D_i \cap D_j}{D_i \cup D_j} \quad [1]$$

where D_i and D_j the datasets (PCHi-C or TAD) corresponding to the hematopoietic cells i and j.

We projected the obtained similarity matrix into a 2D Principal Component Analysis (PCA) space (Supplementary Figure S6).

We can discern a distinct division between lymphoid cells (represented by red dots) and myeloid cells (represented by blue dots) in the PCHi-C fragment PCA and, to a lesser extent, in the TAD PCA. Furthermore,

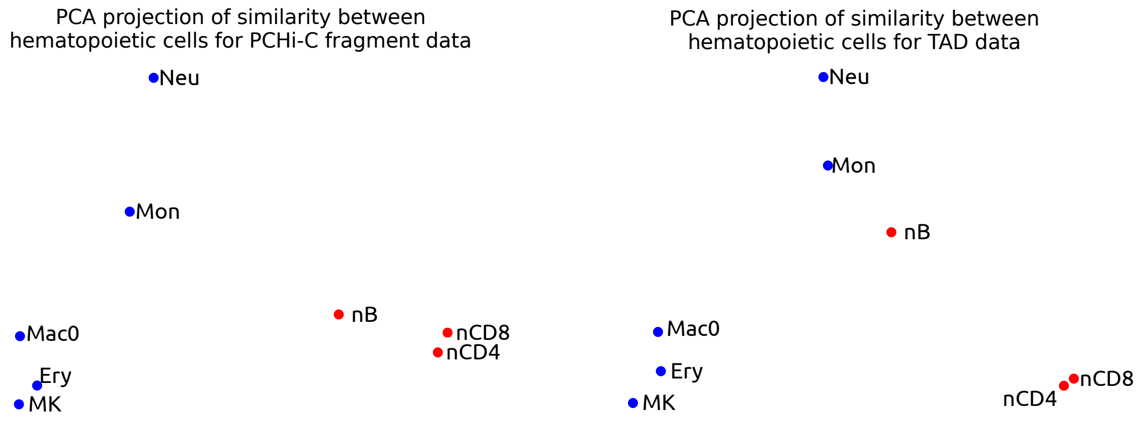

Fig. S6. 2D PCA projection of the Jaccard index similarities between the different hematopoietic cell types. Left: similarities computed on the PCHi-C fragment dataset. Right: similarities computed on the TAD dataset. Red: Lymphoid cells. Blue: Myeloid cells.

on both PCA plots, there is proximity between nCD4 and nCD8 cells, while nB cells are positioned closely to both of these hematopoietic cell types. The Mon and the Neu cell types are also closer to each other than to the other cell types. Finally, the Ery and MK show good proximity in both the PCHi-C fragment and the TAD PCA.

In conclusion, those projections show that both PCHi-C and TAD datasets reflect the tree lineage of the hematopoietic cells.

B. RWR exploration of the multilayer networks including PCHi-C and TAD network layers reflects the tree lineage of the hematopoietic cells.

In the previous section, we demonstrated that the PCHi-C fragments and TAD datasets can effectively capture the tree lineage of hematopoietic cells. We now aim to illustrate that MultiXrank immune diseases diffusion profiles computed on the height hematopoietic multilayer networks, can also capture this lineage.

We used MultiXrank to explore the hematopoietic multilayer networks, using as seeds the 131 different immune diseases iteratively (Supplementary Table S13). Hence, we produced 131 different RWR output scores (one for each immune disease) for each of the 8 hematopoietic multilayer network. In addition, since MultiXrank produces output scores for each of the 4 node types in the multilayer networks (gene/protein nodes, disease nodes, PCHI-C fragment nodes and TAD nodes), we obtained overall 131 (immune disease seeds) $\times$ 8 (cell types) $\times$ 4 (node types) RWR vectors.

Based on these RWR vectors, we defined a similarity measure between the different hematopoietic cell types. First, we compute a disease-disease distance matrix for each node type and hematopoietic network independently. This is done by comparing the rankings of nodes obtained for every pair of immune disease seed (equation 1, Materials and Methods).

To compute cell-cell similarities from those disease-disease distance matrices, we compute the Pearson's correlations coefficients between each disease vector for a specific node type and average it, as detailed below:

$$P^t_{cell_i,cell_j} = \frac{\sum_{d=1}^{N_d} cor(D_d^{cell_i,t}, D_d^{cell_j,t})}{N_d}$$

with $P^t_{cell_i,cell_j}$ the averaged Pearson's correlations for hematopoietic cells i and j for node type t, N_d the total number of diseases, $D_d^{cell_i,t}$ the distance vector of disease d in cell $cell_i$ and node type t, and $D_d^{cell_j,t}$ the distance vector of disease d in cell $cell_j$ and node type t.

This produces one similarity matrix of size 8×8 for each node type (i.e. protein, disease, PCHi-C fragment and TAD) independently.

We projected those 4 node types similarity matrices into a 2D PCA space (Supplementary Figure S7). Those result show that the scores obtained on TAD nodes were the ones that best captured the tree lineage of hematopoietic cells, although the disease and PCHi-C fragment node types have also captured some similarities between nearby cells in the tree lineage. Scores obtained on the protein node type did not allow us to recover the lineage.

Finally, we fuse the cell-cell similarity matrices from the four node types by computing their average Pearson correlations across node types as follow:

$$P^{int}_{cell_i,cell_j} = \frac{\sum_{t=1}^{N_t} cor(P^t_{cell_i}, P^t_{cell_j})}{N_t}$$

with $P^{int}_{i,j}$ the integrated averaged Pearson's correlations for hematopoietic cell $cell_i$ and $cell_j$, N_t the total number of node types in the multilayer network, and with P^t_i (resp. P^t_j) the correlation vector of cell $cell_i$ (resp. $cell_j$) computed for node type t.

The matrix obtained from this procedure represents the similarity of hematopoietic cells based on the integrated MultiXrank scores. We again projected the matrix into a 2D PCA space (Supplementary Figure S8). We observe that the integrated MultiXrank score accurately mirrors the lineage of hematopoietic cells. Indeed, lymphoid cells (red) and myeloid cells (blue) exhibit a separation in the PCA space (PC1). Moreover, nCD4 and nCD8 cells are in close proximity to each other and relative proximity with nB cells. Furthermore, Macrophage (Mac0) and its precursor, Monocyte (Mon), appear closely situated. Additionally, Erythrocyte (Ery) and Megakaryocyte (MK), both stemming from the same progenitor, also exhibit high proximity to each other.

This projection is the one that most effectively summarises the tree lineage, when compared to projecting the similarity matrices of the four node types independently. It demonstrates that MultiXrank is able to capture meaning-full patterns in a multilayer network and reinforces the importance of incorporating different layers in the hematopoietic multilayer networks.

Table S11. MultiXrank parameters used for diffusion profiles comparison to unveil immune diseases similarities.

r	δ	τ	λ	η
0.7	$\begin{bmatrix} 1/2 & 0 & 0 & 0 \end{bmatrix}$	$\begin{bmatrix} 1/3 & 1/3 & 1/3 \\ 1 & 0 & 0 \\ 1 & 0 & 0 \\ 1 & 0 & 0 \end{bmatrix}$	$\begin{bmatrix} 1/4 & 1/4 & 1/4 & 1/4 \\ 1/4 & 1/4 & 1/4 & 1/4 \\ 1/4 & 1/4 & 1/4 & 1/4 \\ 1/4 & 1/4 & 1/4 & 1/4 \end{bmatrix}$	$\begin{bmatrix} 0 & 0 & 0 & 1 \end{bmatrix}$

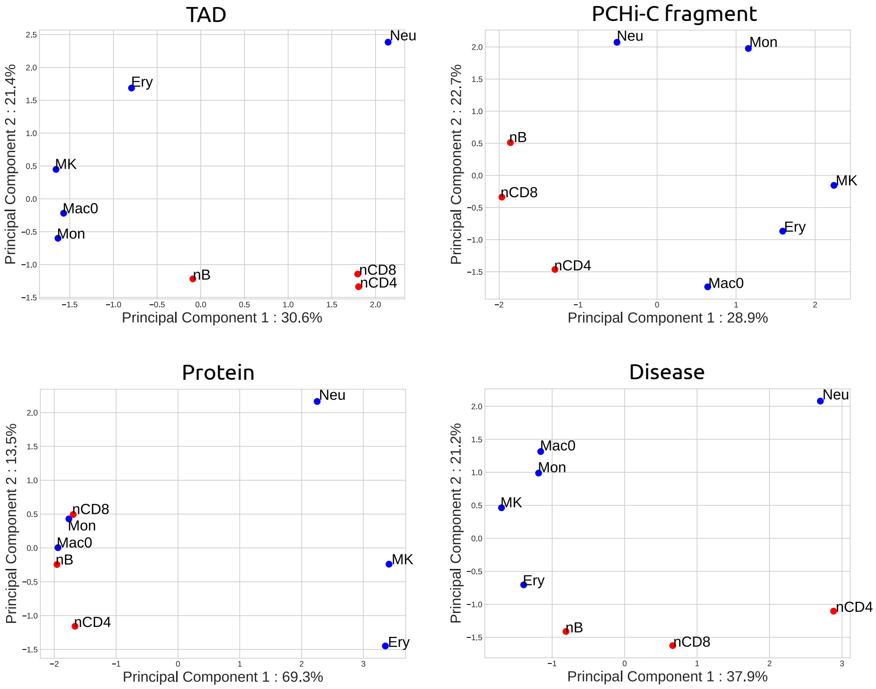

Fig. S7. 2D PCA projection of the similarity between the hematopoietic cell types. The tree lineage of hematopoietic cells is correctly found with the PCHi-C fragment, TAD and disease output scores of MultiXrank. However, the tree lineage is not recovered for the protein output scores.

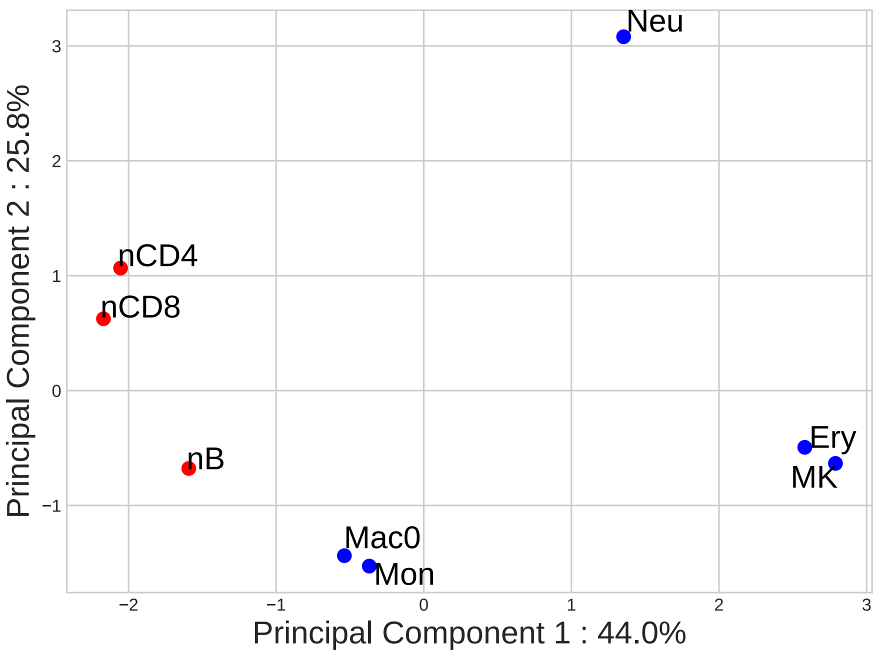

Fig. S8. 2D PCA projection of hematopoietic cell similarities with respect to the integrated MultiXrank output scores effectively visualises the similarity between various hematopoietic cell types. In this projection, lymphoid cells (depicted in red) and myeloid cells (depicted in blue) exhibit a relative separation within the PCA space. Moreover, nCD4 and nCD8 cells are in close proximity to each other and relative proximity with nB cells. Furthermore, Macrophage (Mac0) and its precursor, Monocyte (Mon), appear closely situated. Additionally, Erythrocyte (Ery) and Megakaryocyte (MK), both stemming from the same progenitor, also exhibit high proximity to each other.

Table S12. Composition and characteristics of the immune disease clusters

Cl	Diseases	Characteristics
**0**	Amyloidosis familial visceral ; Autoimmune polyglandular syndrome type 1 ; Autosomal recessive early-onset inflammatory bowel disease 25 ; Autosomal recessive early-onset inflammatory bowel disease 28 ; Behcet's disease ; Blau syndrome ; Chronic recurrent multifocal osteomyelitis (CRMO) ; Cicatricial pemphigoid ; Cryoglobulinemic vasculitis ; Eosinophilic esophagitis (EoE) ; Eosinophilic fasciitis ; Familial cold autoinflammatory syndrome ; Familial Mediterranean fever ; Giant cell arteritis (temporal arteritis) ; Goodpasture's syndrome ; Granulomatosis with Polyangiitis ; Hyper-IgD syndrome ; Kawasaki disease (Mucocutaneous Lymph Node Syndrome) ; Majeed syndrome ; Myasthenia gravis ; Mycosis fungoides ; Myositis ; Netherton syndrome ; Papillon Lefevre syndrome ; Primary biliary cirrhosis ; Pyogenic arthritis, pyoderma gangrenosum and acne ; Rheumatoid arthritis ; Schmidt syndrome ; Takayasu's arteritis ; Tumor necrosis factor receptor-associated periodic syndrome ; Type 1 diabetes	Inflammatory, Autoinflammatory and Autoimmune Diseases.
**1**	Activated PI3K-Delta Syndrome 1 ; Activated PI3K-Delta Syndrome 2 ; Agammaglobulinemia ; Autoimmune lymphoproliferative syndrome ; Autoimmune lymphoproliferative syndrome due to CTLA4 haploinsuffiency ; Autosomal dominant hyper IgE syndrome ; Autosomal recessive candidiasis familial chronic mucocutaneous ; C1q deficiency ; Combined immunodeficiency with skin granulomas ; Complement component 2 deficiency ; Complement component 8 deficiency type 1 ; Complement component 8 deficiency type 2 ; Cyclic neutropenia ; Deficiency of interleukin-1 receptor antagonist ; Dendritic cell, monocyte, B lymphocyte, and natural killer lymphocyte deficiency ; Familial hemophagocytic lymphohistiocytosis ; Felty's syndrome ; Hashimoto's thyroiditis ; Hepatic venoocclusive disease with immunodeficiency ; Hypogammaglobulinemia AGM2 (IGLL1) ; Hypogammaglobulinemia AGM3 (CD79A) ; Hypogammaglobulinemia AGM4 (BLNK) ; Hypogammaglobulinemia AGM5 (LRRC8A) ; Hypogammaglobulinemia AGM6 (CD79B) ; ICF syndrome ; IL12RB1 deficiency ; Immune dysfunction with T-cell inactivation due to calcium entry defect 1 ; Immune dysregulation, polyendocrinopathy and enteropathy X-linked ; Immune thrombocytopenic purpura (ITP) ; Immunodeficiency with hyper IgM type 1 ; Immunodeficiency with hyper IgM type 2 ; Immunodeficiency with hyper IgM type 3 ; Immunodeficiency with hyper IgM type 4 ; Immunodeficiency with hyper IgM type 5 ; Immunodeficiency without anhidrotic ectodermal dysplasia ; Immunoglobulin A deficiency 2 ; Intestinal atresia multiple ; IRAK-4 deficiency ; Isolated growth hormone deficiency type 3 ; Leukocyte adhesion deficiency type 1 ; LRBA deficiency ; MHC class 1 deficiency ; MYD88 deficiency ; Neutrophil-specific granule deficiency ; PGM3-CDG ; Poikiloderma with neutropenia ; Purine nucleoside phosphorylase deficiency ; Reticular dysgenesis ; Severe congenital neutropenia X-linked ; Short-limb skeletal dysplasia with severe combined immunodeficiency ; Sjögren's syndrome ; Spondyloenchondrodysplasia with immune dysregulation ; T-cell immunodeficiency, congenital alopecia and nail dystrophy ; Vici syndrome ; WHIM syndrome ; Wiskott Aldrich syndrome ; X-linked immunodeficiency with magnesium defect, Epstein-Barr virus infection and neoplasia ; X-linked lymphoproliferative syndrome ; ZAP-70 deficiency	Immunodeficiencies, Primary Immunodeficiencies, Increased Susceptibility to Infections
**2**	22q11.2 deletion syndrome (DiGeorge syndrome) ; Acute myeloid leukaemia ; Aicardi-Goutieres syndrome 1 ; Aicardi-Goutieres syndrome 2 ; Asymmetric crying face association ; Ataxia telangiectasia ; Barth syndrome ; Bloom syndrome ; Burkitt lymphoma ; Cartilage-hair hypoplasia ; CHARGE syndrome ; Chediak-Higashi syndrome ; Cherubism ; Chronic lymphocytic leukaemia ; Chronic myelogenous leukaemia ; Congenital heart block ; Epidermodysplasia verruciformis ; Glycogen storage disease type 1B ; Griscelli syndrome type 2 ; Hereditary folate malabsorption ; Hermansky Pudlak syndrome 2 ; Hodgkin's lymphoma ; Inclusion body myositis (IBM) ; Lichen sclerosus ; Melkersson-Rosenthal syndrome ; Meniere's disease ; Myelodysplastic syndromes ; Non-Hodgkin lymphoma ; Osteopetrosis autosomal recessive 7 ; Parry Romberg syndrome ; Pearson syndrome ; Pernicious anemia (PA) ; Pruritic urticarial papules plaques of pregnancy ; Pseudo-TORCH syndrome ; Raynaud's phenomenon ; Schimke immuno-osseous dysplasia ; Shprintzen syndrome ; Shwachman-Diamond syndrome ; Stiff person syndrome (SPS) ; TARP syndrome ; Woods Black Norbury syndrome	Blood-associated diseases (including leukaemia and Lymphoma), Cardiovascular, Dermatological, Hepatic, Muscular, Neurological, Neuromuscular and Skeletal diseases.

Table S13. List of the 131 immune diseases considered in this study. The first column represents the number used to identify each disease in the t-SNE projection. The second column represents the name of the disease. The third column is the UMLS identifier of the disease.

ID	Immune Disease Name	UMLS
0	Agammaglobulinemia	UMLS:C0221026
1	Behcet's disease	UMLS:C0004943
2	Chronic recurrent multifocal osteomyelitis (CRMO)	UMLS:C0410422
3	Cicatricial pemphigoid	UMLS:C1282359
4	Congenital heart block	UMLS:C0149530
5	Eosinophilic esophagitis (EoE)	UMLS:C0341106
6	Eosinophilic fasciitis	UMLS:C0264005
7	Giant cell arteritis (temporal arteritis)	UMLS:C1956391
8	Goodpasture's syndrome	UMLS:C0403529
9	Granulomatosis with Polyangiitis	UMLS:C3495801
10	Hashimoto's thyroiditis	UMLS:C0677607
11	Hypogammaglobulinemia AGM2 (IGLL1)	UMLS:C3150750
12	Hypogammaglobulinemia AGM3 (CD79A)	UMLS:C3150751
13	Hypogammaglobulinemia AGM4 (BLNK)	UMLS:C3150752
14	Hypogammaglobulinemia AGM5 (LRRC8A)	UMLS:C3150753
15	Hypogammaglobulinemia AGM6 (CD79B)	UMLS:C3150207
16	Immune thrombocytopenic purpura (ITP)	UMLS:C0398650
17	Inclusion body myositis (IBM)	UMLS:C0238190
18	Kawasaki disease (Mucocutaneous Lymph Node Syndrome)	UMLS:C0026691
19	Lichen sclerosus	UMLS:C0023652
20	Meniere's disease	UMLS:C0025281
21	Myasthenia gravis	UMLS:C0026896
22	Myositis	UMLS:C0027121
23	Parry Romberg syndrome	UMLS:C0015458
24	Pernicious anemia (PA)	UMLS:C0002892
25	Primary biliary cirrhosis	UMLS:C0008312
26	Raynaud's phenomenon	UMLS:C0034734
27	Rheumatoid arthritis	UMLS:C0003873
28	Schmidt syndrome	UMLS:C0085860
29	Sjögren's syndrome	UMLS:C1527336
30	Stiff person syndrome (SPS)	UMLS:C0085292
31	Takayasu's arteritis	UMLS:C0039263
32	Burkitt lymphoma	UMLS:C0006413
33	Acute myeloid leukaemia	UMLS:C0023467
34	Chronic lymphocytic leukaemia	UMLS:C0023434
35	Chronic myelogenous leukaemia	UMLS:C0023473
36	Hodgkin's lymphoma	UMLS:C0019829
37	Myelodysplastic syndromes	UMLS:C3463824
38	Non-Hodgkin lymphoma	UMLS:C0024305
39	Mycosis fungoides	UMLS:C0026948
40	Type 1 diabetes	UMLS:C0011854

ID	Name	UMLS
41	Shprintzen syndrome	UMLS:C0220704
42	Asymmetric crying face association	UMLS:C0431406
43	22q11.2 deletion syndrome (DiGeorge syndrome)	UMLS:C0012236
44	Pseudo-TORCH syndrome	UMLS:C3489725
45	Aicardi-Goutieres syndrome 1	UMLS:C0796126
46	Aicardi-Goutieres syndrome 2	UMLS:C3489724
47	Amyloidosis familial visceral	UMLS:C0268389
48	Ataxia telangiectasia	UMLS:C0004135
49	Autoimmune lymphoproliferative syndrome	UMLS:C1328840
50	Autoimmune lymphoproliferative syndrome due to CTLA4 haploinsuffiency	UMLS:C4015214
51	Autoimmune polyglandular syndrome type 1	UMLS:C0085859
52	Autosomal dominant hyper IgE syndrome	UMLS:C3489795
53	Autosomal recessive candidiasis familial chronic mucocutaneous	UMLS:C3714992
54	Autosomal recessive early-onset inflammatory bowel disease 28	UMLS:C2751053
55	Autosomal recessive early-onset inflammatory bowel disease 25	UMLS:C2675508
56	Barth syndrome	UMLS:C0574083
57	Blau syndrome	UMLS:C1861303
58	Bloom syndrome	UMLS:C0005859
59	C1q deficiency	UMLS:C3150902
60	Cartilage-hair hypoplasia	UMLS:C0220748
61	CHARGE syndrome	UMLS:C0265354
62	Chediak-Higashi syndrome	UMLS:C0007965
63	Cherubism	UMLS:C0008029
64	Combined immunodeficiency with skin granulomas	UMLS:C2673536
65	Complement component 2 deficiency	UMLS:C3150275
66	Complement component 8 deficiency type 1	UMLS:C3151081
67	Complement component 8 deficiency type 2	UMLS:C3151080
68	Cryoglobulinemic vasculitis	UMLS:C1852456
69	Cyclic neutropenia	UMLS:C0221023
70	Deficiency of interleukin-1 receptor antagonist	UMLS:C2748507
71	Dendritic cell, monocyte, B lymphocyte, and natural killer lymphocyte deficiency	UMLS:C3280030
72	Epidermodysplasia verruciformis	UMLS:C0014522
73	Familial cold autoinflammatory syndrome	UMLS:C0343068
74	Familial hemophagocytic lymphohistiocytosis	UMLS:C0272199
75	Familial Mediterranean fever	UMLS:C0031069
76	Felty’s syndrome	UMLS:C0015773
77	Glycogen storage disease type 1B	UMLS:C0268146
78	Griscelli syndrome type 2	UMLS:C1868679
79	Hepatic venoocclusive disease with immunodeficiency	UMLS:C1856128
80	Hereditary folate malabsorption	UMLS:C0342705

ID	Name	UMLS
81	Hermansky Pudlak syndrome 2	UMLS:C1842362
82	Hyper-IgD syndrome	UMLS:C0398691
83	ICF syndrome	UMLS:C3279748
84	IL12RB1 deficiency	UMLS:C4013949
85	Immune dysfunction with T-cell inactivation due to calcium entry defect 1	UMLS:C2748568
86	Immunodeficiency with hyper IgM type 1	UMLS:C0398689
87	Immunodeficiency with hyper IgM type 2	UMLS:C1720956
88	Immunodeficiency with hyper IgM type 3	UMLS:C1720957
89	Immunodeficiency with hyper IgM type 4	UMLS:C1842413
90	Immunodeficiency with hyper IgM type 5	UMLS:C1720958
91	Immunodeficiency without anhidrotic ectodermal dysplasia	UMLS:C1845117
92	Immune dysregulation, polyendocrinopathy and enteropathy X-linked	UMLS:C0342288
93	Immunoglobulin A deficiency 2	UMLS:C1836032
94	Intestinal atresia multiple	UMLS:C0220744
95	IRAK-4 deficiency	UMLS:C1843256
96	Isolated growth hormone deficiency type 3	UMLS:C0472813
97	Leukocyte adhesion deficiency type 1	UMLS:C0398738
98	LRBA deficiency	UMLS:C3553512
99	Majeed syndrome	UMLS:C1864997
100	Melkersson-Rosenthal syndrome	UMLS:C0025235
101	MHC class 1 deficiency	UMLS:C1858266
102	MYD88 deficiency	UMLS:C2677092
103	Netherton syndrome	UMLS:C0265962
104	Neutrophil-specific granule deficiency	UMLS:C0398593
105	Osteopetrosis autosomal recessive 7	UMLS:C2676766
106	Papillon Lefevre syndrome	UMLS:C0030360
107	Activated PI3K-Delta Syndrome 2	UMLS:C4014934
108	Activated PI3K-Delta Syndrome	UMLS:C3714976
109	Pearson syndrome	UMLS:C0342784
110	PGM3-CDG	UMLS:C4014371
111	Poikiloderma with neutropenia	UMLS:C1858723
112	Pruritic urticarial papules plaques of pregnancy	UMLS:C0269680
113	Purine nucleoside phosphorylase deficiency	UMLS:C0268125
114	Pyogenic arthritis, pyoderma gangrenosum and acne	UMLS:C1858361
115	Reticular dysgenesis	UMLS:C0272167
116	Schimke immuno-osseous dysplasia	UMLS:C0877024
117	Severe congenital neutropenia X-linked	UMLS:C1845987
118	Short-limb skeletal dysplasia with severe combined immunodeficiency	UMLS:C1860168
119	Shwachman-Diamond syndrome	UMLS:C0272170
120	Spondyloenchondrodysplasia with immune dysregulation	UMLS:C1842763

ID	Name	UMLS
121	T-cell immunodeficiency, congenital alopecia and nail dystrophy	UMLS:C1866426
122	TARP syndrome	UMLS:C1839463
123	Tumor necrosis factor receptor-associated periodic syndrome	UMLS:C1275126
124	Vici syndrome	UMLS:C1855772
125	WHIM syndrome	UMLS:C0472817
126	Wiskott Aldrich syndrome	UMLS:C0043194
127	Woods Black Norbury syndrome	UMLS:C1848144
128	X-linked immunodeficiency with magnesium defect, Epstein-Barr virus infection and neoplasia	UMLS:C3275445
129	X-linked lymphoproliferative syndrome	UMLS:C0549463
130	ZAP-70 deficiency	UMLS:C2931299

5. MultiXrank workflow

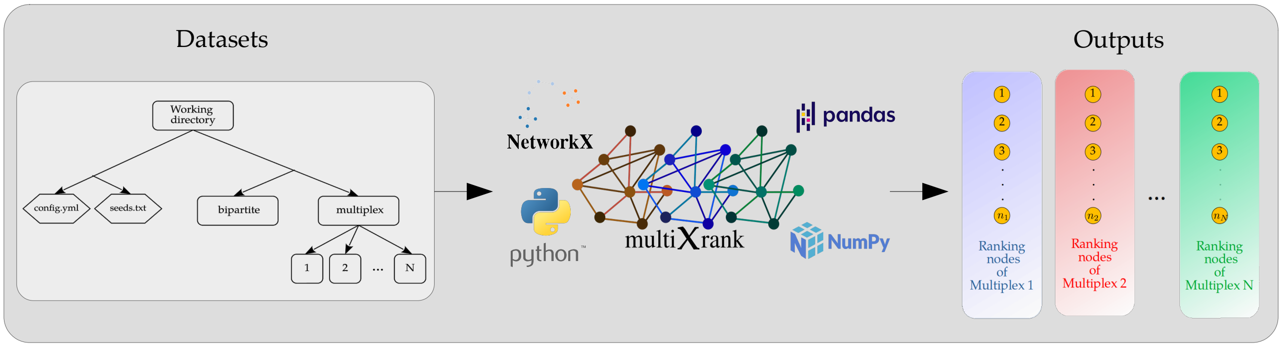

Fig. S9. Workflow of the MultiXrank Python package (39). The networks and the parameters need to be ordered as in the tree file in the left panel. This tree file is used by MultiXrank to run the RWR on the multilayer network defined in the bipartite and multiplex folders with the parameters given in the "config.yml" and "seeds.txt" files. Finally, MultiXrank returns the output scores in an output folder as shown in the right panel, each multiplex/monoplex network is associated with a ranking distribution of the nodes involved in this specific network. A Conda environment is available on GitHub (https://github.com/anthbapt/multixrank) to install all dependencies needed by MultiXrank.

Supplementary References

1. Himmelstein DS, et al. (2017) Systematic integration of biomedical knowledge prioritizes drugs for repurposing. *eLife* 6:e26726.
2. Drew K, et al. (2017) Integration of over 9,000 mass spectrometry experiments builds a global map of human protein complexes. *Mol Syst Biol* 13(6):932.
3. Giurgiu M, et al. (2019) Corum: the comprehensive resource of mammalian protein complexes–2019. *Nucleic Acids Res* 47(D1):D559–D563.
4. Türei D, et al. (2021) Integrated intra- and intercellular signaling knowledge for multicellular omics analysis. *Molecular Systems Biology* 17(3):e9923.
5. Pratt D, et al. (2015) Ndex, the network data exchange. *Cell Systems* 1(4):302–305.
6. Croft D, et al. (2014) The reactome pathway knowledgebase. *Nucleic Acids Res* 42(D1):D472–D477.
7. Köhler S, et al. (2021) The Human Phenotype Ontology in 2021. *Nucleic Acids Research* 49(D1):D1207–D1217.
8. Valdeolivas A, et al. (2018) Random walk with restart on multiplex and heterogeneous biological networks. *Bioinformatics* 35(3):497–505.
9. Wishart DS, et al. (2018) DrugBank 5.0: a major update to the DrugBank database for 2018. *Nucleic Acids Research* 46(D1):D1074–D1082.
10. Cheng F, Kovács IA, Barabási AL (2019) Network-based prediction of drug combinations. *Nature Communications* 10(1):1197.
11. Javierre BM, et al. (2016) Lineage-specific genome architecture links enhancers and non-coding disease variants to target gene promoters. *Cell* 167(5):1369–1384.e19.
12. Piñero J, et al. (2015) Disgenet: a discovery platform for the dynamical exploration of human diseases and their genes. *Database (Oxford)* 2015.
13. Piñero J, et al. (2020) The disgenet knowledge platform for disease genomics: 2019 update. *Nucleic Acids Res* 48(D1):D845–D855.
14. Brown AS, Patel CJ (2017) A standard database for drug repositioning. *Scientific Data* 4(1):170029.
15. Frankish A, et al. (2021) GENCODE 2021. *Nucleic Acids Research* 49(D1):D916–D923.
16. Cairns J, et al. (2016) Chicago: robust detection of dna looping interactions in capture hi-c data. *Genome Biology* 17(1):127.
17. Dixon JR, et al. (2012) Topological domains in mammalian genomes identified by analysis of chromatin interactions. *Nature* 485(7398):376–380.
18. Fang L, et al. (2021) GRNdb: decoding the gene regulatory networks in diverse human and mouse conditions. *Nucleic Acids Research* 49(D1):D97–D103.
19. Li Y, et al. (2021) Single-cell transcriptomic analysis reveals dynamic alternative splicing and gene regulatory networks among pancreatic islets. *Science China. Life Sciences* 64(1):174–176.
20. Robinson JL, et al. (2020) An Atlas of Human Metabolism. *Science signaling* 13(624):eaaz1482.
21. Felix CA, et al. (1998) Association of CYP3A4 genotype with treatment-related leukemia. *Proceedings of the National Academy of Sciences* 95(22):13176–13181. Publisher: Proceedings of the National Academy of Sciences.
22. Kobayashi T, et al. (2010) Molecular and clinical analysis of RAF1 in Noonan syndrome and related disorders: dephosphorylation of serine 259 as the essential mechanism for mutant activation. *Human Mutation* 31(3):284–294.
23. Hasle H (2009) Malignant Diseases in Noonan Syndrome and Related Disorders. *Hormone Research* 72(Suppl. 2):8–14.
24. Oki T, et al. (2012) Aberrant expression of RasGRP1 cooperates with gain-of-function NOTCH1 mutations in T-cell leukemogenesis. *Leukemia* 26(5):1038–1045. Number: 5 Publisher: Nature Publishing Group.
25. Li H, et al. (2019) Knockdown of diacylglycerol kinase zeta (DGKZ) induces apoptosis and G2/M phase arrest in human acute myeloid leukemia HL-60 cells through MAPK/survivin/caspase pathway. *Die Pharmazie - An International Journal of Pharmaceutical Sciences* 74(7):418–422.
26. Trino S, et al. (2016) Inverse regulation of bridging integrator 1 and BCR-ABL1 in chronic myeloid leukemia. *Tumor Biology* 37(1):217–225.
27. Xu Y, Wertheim G, Morrissette JJD, Bagg A (2017) Braf kinase domain mutations in de novo acute

myeloid leukemia with monocytic differentiation. *Leukemia & Lymphoma* 58(3):743–745. PMID: 27545333.
28. Zhang Y, et al. (2020) Role of RASA1 in cancer: A review and update (Review). *Oncology Reports* 44(6):2386–2396.
29. Lucena-Araujo AR, et al. (2011) High expression of AURKA and AURKB is associated with unfavorable cytogenetic abnormalities and high white blood cell count in patients with acute myeloid leukemia. *Leukemia Research* 35(2):260–264.
30. Lee JW, et al. (2005) Mutational analysis of the ARAF gene in human cancers. *APMIS* 113(1):54–7. _eprint: https://onlinelibrary.wiley.com/doi/pdf/10.1111/j.1600-0463.2005.apm1130108.x.
31. Laverdière I, et al. (2018) Leukemic stem cell signatures identify novel therapeutics targeting acute myeloid leukemia. *Blood Cancer Journal* 8(6):1–16. Number: 6 Publisher: Nature Publishing Group.
32. Lethaby C, et al. (2007) Bisphosphonate Therapy for Reduced Bone Mineral Density During Treatment of Acute Lymphoblastic Leukemia in Childhood and Adolescence: A Report of Preliminary Experience. *Journal of Pediatric Hematology/Oncology* 29(9):613.
33. Auclair D, et al. (2007) Antitumor activity of sorafenib in FLT3-driven leukemic cells. *Leukemia* 21(3):439–445. Number: 3 Publisher: Nature Publishing Group.
34. Fan RF, et al. (2016) Zoledronic acid overcomes adriamycin resistance in acute myeloid leukemia cells by promoting apoptosis. *Molecular Medicine Reports* 14(6):5660–5666. Publisher: Spandidos Publications.
35. Fiedler W, et al. (2015) A phase I/II study of sunitinib and intensive chemotherapy in patients over 60 years of age with acute myeloid leukaemia and activating FLT3 mutations. *British Journal of Haematology* 169(5):694–700. _eprint: https://onlinelibrary.wiley.com/doi/pdf/10.1111/bjh.13353.
36. Kessler T, et al. (2019) Phase II clinical trial of pazopanib in patients with acute myeloid leukemia (AML), relapsed or refractory or at initial diagnosis without an intensive treatment option (PazoAML). *Annals of Hematology* 98(6):1393–1401.
37. Lainey E, et al. (2012) Erlotinib antagonizes ABC transporters in acute myeloid leukemia. *Cell Cycle* 11(21):4079–4092. Publisher: Taylor & Francis _eprint: https://doi.org/10.4161/cc.22382.
38. Martinez FO, Gordon S, Locati M, Mantovani A (2006) Transcriptional profiling of the human monocyte-to-macrophage differentiation and polarization: New molecules and patterns of gene expression. *The Journal of Immunology* 177(10):7303–7311.
39. Baptista A, Gonzalez A, Baudot A (2022) Universal multilayer network exploration by random walk with restart. *Communications Physics* 5(1):170.
